# Simplified dosing of oral azithromycin for children 1–11 months old in child survival programmes: age-based and height-based dosing protocols

**DOI:** 10.1136/bmjgh-2022-009801

**Published:** 2022-10-17

**Authors:** Huiyu Hu, Ahmed Mamane Arzika, Ali Sie, Amza Abdou, Ramatou Maliki, Alio Karamba Mankara, Mamadou Outtara, Mamadou Bountogo, Valentin Boudo, Fanny Yago-Wienne, Issouf Bamba, Charles Knirsch, Paul Emerson, PJ Hooper, Elodie Lebas, Jessica Brogdon, Fanice Nyatigo, Catherine E Oldenburg, Thomas M Lietman, Kieran S O'Brien

**Affiliations:** 1 Francis I. Proctor Foundation, University of California, San Francisco, California, USA; 2 Centre de Recherche et Interventions en Sante Publique, Niamey, Niger; 3 Centre de Recherche en Sante de Nouna, Nouna, Burkina Faso; 4 Programme Nationale de Santé Oculaire, Niamey, Niger; 5 Helen Keller International, Ouagadougou, Burkina Faso; 6 Pfizer Inc, New York, New York, USA; 7 International Trachoma Initiative, Decatur, Georgia, USA; 8 Department of Epidemiology and Biostatistics, University of California, San Francisco, California, USA; 9 Department of Ophthalmology, University of California, San Francisco, California, USA; 10 Institute for Global Health Sciences, University of California, San Francisco, California, USA

**Keywords:** child health, public health

## Abstract

**Background:**

To facilitate mass distribution of azithromycin, trachoma control programmes use height instead of weight to determine dose for children 6 months to 15 years old. WHO has recommended azithromycin distribution to children 1–11 months old to reduce mortality in high mortality settings under carefully monitored conditions. Weight was used to determine dose in children 1–5 months old in studies of azithromycin distribution for child survival, but a simplified approach using age or height for all aged 1–11 months old could increase programme efficiency in real-world settings.

**Methods:**

This secondary analysis used data from two cluster randomised trials of azithromycin distribution for child mortality in Niger and Burkina Faso. An exhaustive search algorithm was developed to determine the optimal dose for different age groups, using tolerance limits of 10–20 mg/kg for children 1–2 months old and 15–30 mg/kg for children 3–11 months old. Height-based dosing was evaluated against the existing trachoma dosing pole and with a similar exhaustive search.

**Results:**

The optimal two-tiered age-based approach suggested a dose of 80 mg (2 mL) for children 1–2 months old and 160 mg (4 mL) for children 3–11 months old. Under this schedule, 89%–93% of children would have received doses within tolerance limits in both study populations. Accuracy was 93%–94% with a three-tiered approach, which resulted in doses of 80 mg (2 mL), 120 mg (3 mL) and 160 mg (4 mL) for children 1–2, 3–4 and 5–11 months old, respectively. For children 1–5 months old, the existing height pole would result in 70% of doses within tolerance limits. The optimisation identified height-based dosing options with 95% accuracy, although this would require changes to the existing dosing pole as well as additional training to measure infants lying flat.

**Conclusions:**

Overall, an age-based approach with two age tiers resulted in high accuracy while considering both concerns about overdosing in this young population and simplicity of field operations.

**WHAT IS ALREADY KNOWN ON THIS TOPIC**

- Azithromycin mass drug administration (MDA) is a core component of trachoma control programmes, which use height to determine dose in children aged 6 months to 15 years.
- Azithromycin MDA to children 1–11 months of age is also being considered as an approach to reduce child mortality, although weight has been used to determine dose in children <6 months in studies of this intervention as limited data have been available to simplify dosing for this age group.
- Programmes considering this intervention could increase speed and efficiency by considering simplified dosing approaches based on age or height instead of weight.

**WHAT THIS STUDY ADDS**

- This study used age, weight and height data from cluster randomised trials of azithromycin MDA for mortality in Niger and Burkina Faso to determine optimal dosing using age and height for children 1–11 months old.
- We identified age-based approaches with >90% accuracy, finding similar high accuracy using two or three age tiers.
- The existing trachoma programme height pole was less accurate in this age group, although changes to the height pole could improve accuracy and allow height to be used for all ages.

**HOW THIS STUDY MIGHT AFFECT RESEARCH, PRACTICE OR POLICY**

- We identified age-based and height-based dosing approaches for azithromycin MDA targeting children 1–11 months old that limit underdosing and overdosing.
- The simplest approach may be to provide a dose of 80 mg (2 mL) to children 1–2 months old and 160 mg (4 mL) to children 3–11 months old.
- As this intervention moves from trial to programme settings, these results present options to improve the efficiency of field operations while ensuring accurate dosing.

## Introduction

Trachoma control programmes provide annual oral azithromycin to everyone ≥6 months old in endemic areas worldwide according to WHO recommendations.^1^ For children, the recommended dosing is 20 mg/kg of bodyweight up to the adult dose of 1 g and all children younger than 7 years old receive oral suspension (40 mg/mL).^2^ However, weight-based dosing involves the use of scales, which can impede programme efficiency. Mass treatment programmes require large numbers of scales that must be calibrated regularly and kept in working order, resulting in substantial supply costs and possible delays if working scales are not available. To facilitate large-scale community-based implementation of azithromycin distributions for trachoma, height-based dosing for children aged 6 months to 15 years was developed. Several studies validated the use of height as a proxy for weight, finding that 90%–98% of children would receive a dose within accepted tolerance limits with proposed height-based dosing strategies.^3–6^ Simplified height-based dosing protocols for children are now universally used in trachoma programmes, typically involving a height pole made of wood or a plastic tape that demarcates the doses associated with ranges of heights.^2^

WHO released conditional guidelines on mass distribution of azithromycin to children 1–11 months of age to improve child survival in high mortality settings in sub-Saharan Africa.^7^ The evidence for these guidelines came in part from randomised controlled trials that demonstrated that mass azithromycin distribution reduced mortality in children 1–59 months of age in some settings.^8 9^ The trials evaluating azithromycin distribution for child mortality used weight-based dosing for children 1–5 months old or unable to stand while following the height-based protocols developed for trachoma programmes for the older children.^8^ The earlier trachoma studies on height-based dosing lacked data to validate a simplified protocol for children <6 months old, since trachoma programmes do not treat this age group with azithromycin as they are not included in regulatory approvals. As azithromycin distribution to children 1–11 months of age is being considered for child survival programmes,^7^ simplified dosing protocols without scales for this age group would reduce the resources and time required to determine dose and deliver the intervention in programme settings. Here, we aimed to evaluate age-based and height-based strategies to determine azithromycin dose in children 1–11 months old as alternatives to weight-based dosing using data from two cluster randomised trials of azithromycin distribution for child mortality in Niger and Burkina Faso.

## Methods

### Data sources: design, setting, participants, variables

This secondary analysis used existing data collected as part of two cluster randomised trials evaluating the biannual distribution of oral azithromycin to children 1–59 months of age in Niger and Burkina Faso.

#### Macrolides Oraux pour le Réduire des Décès avec un Oeil sur la Résistance trial

The Macrolides Oraux pour le Réduire des Décès avec un Oeil sur la Résistance (oral macrolides for the reduction of mortality with an eye on resistance, MORDOR I) mortality trial was conducted in Malawi, Niger and Tanzania from 2014 to 2017.^8^ This analysis includes data from the Niger site of the trial. The Niger site enrolled communities with populations between 200 and 2000 inhabitants in the Boboye and Loga districts in Niger. Eligible communities were randomised to receive biannual distribution of a single 20 mg/kg dose of oral azithromycin or matching placebo to children 1–59 months old weighing at least 3.8 kg. A population-based census was used to enumerate the eligible population, administer azithromycin or placebo and monitor vital status every 6 months during the study period. For children able to stand, dose was determined via the height-based dosing pole used in Niger’s trachoma programme. For children unable to stand, dose was determined by weight which was measured by a hanging scale (American Weigh Scale Amw-tl440, Cumming, Georgia, USA). Overall, 594 communities in Niger were included in the trial. All data were collected electronically using a mobile application (Conexus, Los Gatos, California, USA).

A parallel trial was conducted in 30 communities at the Niger site to monitor additional outcomes separate from the mortality trial (MORDOR morbidity trial).^10^ Eligibility, randomisation and census data collection were the same in both trials, as described above. In addition, repeated random samples of 40 children aged 1–59 months were selected at baseline, 6 months, 12 months and 24 months for additional monitoring. A separate sample of 80 children 1–59 months old was randomly selected before the baseline monitoring visit to be monitored longitudinally. The present study includes data on age, weight and height collected from these samples during the MORDOR morbidity trial. Age was determined based on the date of birth as recorded in the child’s health card or reported by the caregiver, or by age in months or years as reported by the caregiver if date of birth was unavailable. Weight was measured with a Seca 874 floor scale (Seca, Hamburg, Germany) and length or height was measured using a portable stadiometer (Schorr Productions, Olney, Maryland, USA). Measurements were taken in triplicate and the median used for analyses.

#### Child Health with Azithromycin Treatment trials

The Child Health with Azithromycin Treatment (CHAT) trial is conducted in 280 rural communities in the Nouna District in Burkina Faso and is currently ongoing at the time of writing.^11^ The present study included data from baseline visit of the cluster randomised trial included in the CHAT project from August 2019 through January 2020. CHAT used a similar approach to randomisation, treatment and census data collection as described above for MORDOR. In CHAT, the ADE M1116000 hanging scale (Hamburg, Germany) was used to determine weight and data were collected electronically using Survey Solutions (World Bank Group, Washington, District of Columbia, USA). This analysis used data collected on child age and weight as recorded during the census with a hanging scale (procedures described above).

This analysis included children 1–11 months old included in both trials who had both age and weight data available. Children with age <1 month or >11 months or weight-for-age Z-scores (WAZ) <−5 or >6 were excluded. WAZ was calculated according to the 2006 WHO Child Growth Standards using the ‘anthro’ package in R (R Foundation for Statistical Computing, Vienna, Austria) and cutoffs for eligibility were chosen based on WHO recommendations.^12 13^ To evaluate height-based dosing, additional analyses included children 1–5 months old with age and height measurement available from the MORDOR morbidity trial. This age group was chosen because the existing height-based dosing protocols include children ≥6 months old. Age rounded to the nearest month was used for all analyses with the following categorisation: 1 month=31–60 days, 2 months=61–90 days, 3 months=91–121 days, 4 months=122–151 days, 5 months=152–182 days, 6 months=183–212 days, 7 months=213–243 days, 8 months=244–273 days, 9 months=274–303 days, 10 months=304–334 days and 11 months=335–364 days.

### Azithromycin dosing and tolerance limits

Both trials used the paediatric dose of 20 mg/kg as outlined for trachoma programmes.^2^ Prior trachoma studies evaluating height as a proxy for weight used tolerance limits of 15–30 mg/kg, 20–30 mg/kg and 20–40 mg/kg to define underdosing and overdosing based on safety and tolerance data for azithromycin available at the time.^3–6^ Since then, controlled safety and efficacy data of a single dose of 60 mg/kg compared with 30 mg/kg has led to regulatory review and approval of the higher dose of the sustained release formulation of azithromycin for children 6 months to 6 years of age.^14^ In this analysis, we chose tolerance limits of 10–20 mg/kg for children 1–2 months old given the potential macrolide-associated risk of infantile hypertrophic pyloric stenosis (IHPS) in the youngest children. As observational evidence suggests that this risk is highest in the first 2 weeks of life,^15–18^ the MORDOR and CHAT trials excluded children <1 month of age. Some studies indicate this risk might decrease but persist after 1 month of age,^15–17^ thus we chose to exclude protocols that allow for overdosing in children 1 or 2 months old. For children 3–11 months old, we chose the more conservative tolerance limit of 15–30 mg/kg and used 20–40 mg/kg tolerance limits in sensitivity analyses.

### Analysis methods

Descriptive statistics of participants included from each trial were summarised using frequencies and percentages for categorical variables as well as means and SD for continuous variables. Analyses to evaluate age-based dosing were conducted using both the MORDOR morbidity and CHAT datasets. These analyses aimed to select a schedule that maximised the number of children receiving doses within tolerance limits. An exhaustive search algorithm was used to compare each option against the tolerance limits to determine the dose and/or age cut-off with the greatest accuracy. For children 1–2 months old, the optimal dose was selected from 40 to 160 mg (1–4 mL), in increments of 40 mg (1 mL). For children 3–11 months old, two analyses were conducted. The first selected one optimal dose from the range 80–320 mg (2–8 mL), in increments of 40 mg (1 mL). The second analysis first selected the optimal age cut-off by examining cutoffs from the range 3–11 months in 1-month increments and then selected the optimal dose for each age subgroup using the same approach described above. In all scenarios, only integer-based doses were considered in order to increase simplicity of dosing in field settings and align with the existing height-based approach. Ranges of doses to be considered were determined based on the distribution of doses received for the respective age groups in the main MORDOR trial.

Accuracy was used to determine optimal dose by dividing each dose by recorded weight to obtain a dose in mg/kg and comparing the dose against the tolerance limits. The optimal dose was determined to be that which resulted in the greatest percentage of children receiving doses within the tolerance limits as described above. Sensitivity analyses examined tolerance limits of 20–40 mg/kg.

Height was considered as well using the MORDOR morbidity dataset. The accuracy of height-based dosing using the existing Niger trachoma programme dosing pole was evaluated first by comparing the doses a participant would have received using the height pole against the tolerance limits. The second approach used an exhaustive search algorithm to determine the optimal age and height cutoffs for children 1–5 months old. Tolerance limit of 15–30 mg/kg were used in height analyses. Sensitivity analyses included using tolerance limits of 20–40 mg/kg.

### Patient and public involvement and dissemination

Patients and/or the public were not involved in the design, conduct or reporting of this research. The results will be disseminated to community, district, regional and national leaders involved in the implementation of child survival programmes.

## Results

Table 1 summarises characteristics of study participants included from each trial. A total of 4136 and 6408 children 1–11 months old were identified from the MORDOR morbidity and CHAT trials, respectively. From each dataset, children were excluded for having WAZ <−5 or >6 (33 (0.8%) from MORDOR morbidity and 50 (0.8%) from CHAT). The final analyses included 4103 children from MORDOR morbidity and 6358 children from CHAT. Median age was 7 months (IQR 4–9 months) in MORDOR and 6 months (IQR 3–8 months) in CHAT. Mean WAZ was −0.7 (SD 1.4) in MORDOR and −0.9 (SD 1.5 in CHAT), with 51% female participants in MORDOR and 50% in CHAT. In MORDOR, the majority of participants 1–5 months old (74.2%) had a height in the 54 to <65 cm range on the dosing pole, which corresponds to a dose of 160 mg (4 mL).

**Table 1:** Characteristics of included participants 1–11 months old from the MORDOR and CHAT trials

Characteristic	MORDOR morbidity		CHAT	
	N or mean	%* or SD	N or mean	%* or SD
Total				
	4103	100%	6358	100%
Age group, months (n, %)†				
1	142	3.5%	574	9.0%
2	248	6.0%	617	9.7%
3	339	8.3%	559	8.8%
4	319	7.8%	543	8.5%
5	381	9.3%	670	10.5%
6	350	8.5%	612	9.6%
7	434	10.6%	640	10.1%
8	430	10.5%	609	9.6%
9	490	11.9%	567	8.9%
10	513	12.5%	485	7.6%
11	457	11.1%	482	7.6%
Female sex (n, %)	2100	51.2%	3111	48.9%
Weight, kg (mean, SD)	7.3	1.5	6.7	1.5
WAZ, SD (mean, SD)	−0.7	1.4	−0.9	1.5
Height range (corresponding dose)‡			NA	NA
<54 cm (80 mg/2 mL)	106	7.3%	NA	NA
54 to <65 cm (160 mg/4 mL)	1079	74.2%	NA	NA
65 to <76 cm (240 mg/6 mL)	245	16.9%	NA	NA
76 to <87 cm (320 mg/8 mL)	20	1.4%	NA	NA
≥87 cm (400 mg/10 mL)	4	0.3%	NA	NA

*Percentages may not sum to 100% due to rounding.

†Age groups in month correspond to the following age in days: 1 month=31–60 days, 2 months=61–90 days, 3 months=91–121 days, 4 months=122–151 days, 5 months=152–182 days, 6 months=183–212 days, 7 months=213–243 days, 8 months=244–273 days, 9 months=274–303 days, 10 months=304–334 days and 11 months=335–364 days.

‡Using length/height as measured during anthropometry data collection and ranges with doses according to the Niger trachoma programme dosing pole for children 1–5 months of age only (n=1454); not available for CHAT.

CHAT, Child Health with Azithromycin Treatment; MORDOR, Macrolides Oraux pour le Réduire des Décès avec un Oeil sur la Résistance; NA, not available; WAZ, weight-for-age Z-score.

**Figure 1:**
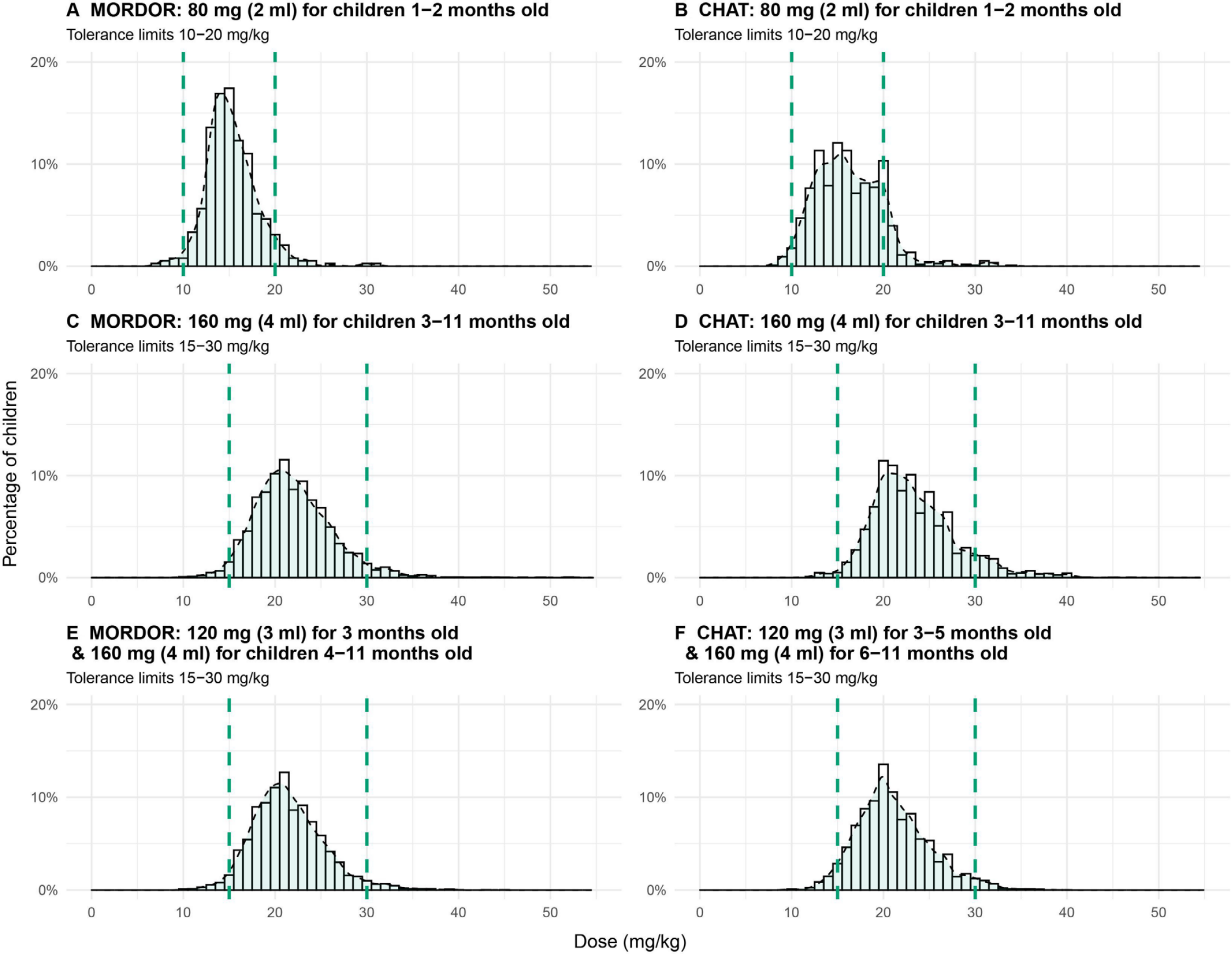
Distribution of results for doses providing the greatest accuracy for children 1–11 months old in the MORDOR and CHAT trials. A and B show results for children 1–2 month of age in MORDOR morbidity and CHAT, respectively. C and D show results for children 3–11 months of age in MORDOR morbidity and CHAT, respectively, with no age cut-off determination. E and F show the results for children 3–11 months of age in MORDOR morbidity and CHAT with age cut-off determination. Green dashed lines indicate the tolerance limits used for each analysis and the dashed line with green shading underneath shows the kernel density estimate for the histogram. CHAT, Child Health with Azithromycin Treatment; MORDOR, Macrolides Oraux pour le Réduire des Décès avec un Oeil sur la Résistance.

Tables 2 and 3 and figure 1 summarise the results of the optimisation analysis to determine dose for each age group from the MORDOR morbidity and CHAT trials. Using tolerance limits of 10–20 mg/kg for participants 1–2 month of age, both trials found the highest accuracy with a dose of 80 mg (2 mL). Accuracy with this dose was 93.3% in MORDOR morbidity and 88.0% in CHAT, with average doses of 15.3 mg/kg and 16.2 mg/kg, respectively.

**Table 2:** Dose optimisation for children 1–11 months old in the MORDOR and CHAT trials, without age cut-off determination

Age group (tolerance limit)	Study (n)	Dose	Within limits		Underdose		Overdose		Average dose (mg/kg)
			N	%	N	%	N	%	
1–2 months (10–20 mg/kg)	MORDOR (390)	40 mg (1 mL)	24	6.2	366	93.8%	NA	NA	7.7
		80 mg (2 mL)	364	93.3	7	1.8%	19	4.9	15.3
		120 mg (3 mL)	96	24.6	NA	NA	294	75.4	22.9
		160 mg (4 mL)	8	2.1	NA	NA	382	97.9	30.6
	CHAT (1191)	40 mg (1 mL)	204	17.1	987	82.9%	NA	NA	8.1
		80 mg (2 mL)	1048	88.0	21	1.8%	122	10.2	16.2
		120 mg (3 mL)	305	25.6	NA	NA	886	74.4	24.3
		160 mg (4 mL)	26	2.2	NA	NA	1165	97.8	32.4
3–11 months (15–30 mg/kg)	MORDOR (3713)	80 mg (2 mL)	160	4.3	3553	95.7%	NA	NA	11.0
		120 mg (3 mL)	2517	67.8	1186	31.9%	10	0.3	16.5
		160 mg (4 mL)	3470	93.5	83	2.2%	160	4.3	22.1
		200 mg (5 mL)	2678	72.1	11	0.3%	1024	27.6	27.6
		240 mg (6 mL)	1297	34.5	1	0.0%	2415	65.0	33.1
		280 mg (7 mL)	348	9.4	NA	NA	3365	90.6	38.6
		320 mg (8 mL)	83	2.2	NA	NA	3630	97.8	44.1
	CHAT (5167)	80 mg (2 mL)	469	9.1	4698	90.9%	NA	NA	11.7
		120 mg (3 mL)	4078	78.9	1080	20.9%	9	0.2	17.5
		160 mg (4 mL)	4637	89.7	61	1.2%	469	9.1	23.4
		200 mg (5 mL)	3210	62.1	3	0.1%	1954	37.8	29.2
		240 mg (6 mL)	1344	26.0	NA	NA	3823	74.0	35.0
		280 mg (7 mL)	243	4.7	NA	NA	4924	95.3	40.8
		320 mg (8 mL)	61	1.2	NA	NA	5106	98.8	46.7

Result with highest accuracy is highlighted for each set of analyses.*

*Percentages may not sum to 100% due to rounding.

CHAT, Child Health with Azithromycin Treatment; MORDOR, Macrolides Oraux pour le Réduire des Décès avec un Oeil sur la Résistance; NA, not available.

**Table 3:** Top five results from dose optimisation for children 3–11 months old in the MORDOR and CHAT trials, with age cut-off determination and tolerance limits 15–30 mg/kg

Study (n)	Age cut-off (months)†	Dose 1, younger group	Dose 2, older group	Within limits		Underdose		Overdose	
				N	%	N	%	N	%
MORDOR (3713)	3	120 mg (3 mL)	160 mg (4 mL)	3507	94.5	98	2.6	108	2.9
	4	120 mg (3 mL)	160 mg (4 mL)	3500	94.3	132	3.6	81	2.2
	3	160 mg (4 mL)	160 mg (4 mL)	3470	93.5	83	2.2	160	4.3
	4	160 mg (4 mL)	160 mg (4 mL)	3470	93.5	83	2.2	160	4.3
	5	120 mg (3 mL)	160 mg (4 mL)	3470	93.5	194	5.2	49	1.3
CHAT (5167)	5	120 mg (3 mL)	160 mg (4 mL)	4839	93.7	186	3.6	142	2.7
	4	120 mg (3 mL)	160 mg (4 mL)	4836	93.6	120	2.3	211	4.1
	6	120 mg (3 mL)	160 mg (4 mL)	4803	93.0	272	5.3	92	1.8
	3	120 mg (3 mL)	160 mg (4 mL)	4785	92.6	81	1.6	301	5.8
	7	120 mg (3 mL)	160 mg (4 mL)	4706	91.1	403	7.8	58	1.1

Result with highest accuracy is highlighted for each set of analyses.*

*Percentages may not sum to 100% due to rounding.

†Age cutoffs refer to the age at which the younger group ends. An age cut-off of 3 indicates a younger group of 3 months old and an older group of 4–11 months old, an age cut-off of 4 indicates a younger group of 3–4 months old and an older group of 5–11 months old, an age cut-off of 5 indicates a younger group of 3–5 months old and an older group of 6–11 months old.

CHAT, Child Health with Azithromycin Treatment; MORDOR, Macrolides Oraux pour le Réduire des Décès avec un Oeil sur la Résistance.

For the older age group, a tolerance limit of 15–30 mg/kg was used. Two sets of analyses were conducted for each trial, the first to select one dose for the entire group of children 3–11 months old and the second to select both dose and an age cut-off to delineate doses for two age groups among children 3–11 months old. In analyses to determine one dose, both MORDOR and CHAT analyses selected a dose of 160 mg (4 mL) for children 3–11 months old with 93.5% and 89.7% accuracy, respectively (table 3, figure 1). When selection for an age cut-off was included in the optimisation, the top results from both trials suggested a dose of 120 mg (3 mL) for the younger age group (3 months in MORDOR and 3–5 months in CHAT) and 160 mg (4 mL) for older age group (4–11 months in MORDOR and 6–11 months in CHAT), resulting in 94% accuracy in both MORDOR and CHAT. Similar results were found in both trials with an age cut-off of 4 months, suggesting a dose of 120 mg (3 mL) for children 3–4 months old and 160 mg (4 mL) for children 5–11 months old. All age cutoffs >5 months resulted in a dose of 4 mL for both younger and older age groups. Heatmaps displaying the full results for the MORDOR and CHAT optimisations to determine both age cut-off and dose are included in online supplemental figures 1 and 2. Sensitivity analyses using tolerance limits of 20–40 mg/kg for children 3–11 months old resulted in higher suggested doses of 200 mg (5 mL) for one age group (online supplemental table 1) or 200–240 mg (5–6 mL) for two age groups, with similar accuracy to the main analyses in both trials. Table 4 proposes dosing schedules based on these results and shows the overall accuracy that would be seen if these approaches were used in each dataset.

10.1136/bmjgh-2022-009801.supp2 Supplementary data

**Table 4:** Proposed age-based dosing schedules for children 1–11 months old based on results from the MORDOR morbidity and CHAT trials*

Tiers	Age group (months)	Dose	% Within limits	
			MORDOR (n=4103)	CHAT (n=5498)
2	1–2	80 mg (2 mL)	93.4%	89.4%
	3–11	160 mg (4 mL)		
3	1–2	80 mg (2 mL)	94.2%	92.5%
	3–4	120 mg (3 mL)		
	5–11	160 mg (4 mL)		

*Assuming tolerance limits of 10–20 mg/kg for children 1–2 months old and 15–30 mg/kg for children 3–11 months old.

CHAT, Child Health with Azithromycin Treatment; MORDOR, Macrolides Oraux pour le Réduire des Décès avec un Oeil sur la Résistance.

Data from the MORDOR morbidity trial were used to evaluate height as an alternative to age in dose determination for children 1–5 months old. The first approach used the existing Niger trachoma programme dosing pole and assumed tolerance limits of 15–30 mg/kg, resulting in 70.0% accuracy (online supplemental figure 3). The second approach used an algorithm to determine the optimal age and height cutoffs to use to determine dose. This approach found the highest accuracy (93.9%) with a dose of 120 mg (3 mL) for children ≤60 cm and 160 mg (4 mL) for children >60 cm (online supplemental table 2). Sensitivity analyses using the higher tolerance limits of 20–40 mg/kg resulted in higher suggested dosing for each group (160 mg (4 mL) for children ≤60 cm and 200 mg (5 mL) for children >60 cm (online supplemental table 2).

## Discussion

As targeted azithromycin distribution is considered for inclusion in child survival efforts, a simplified age-based or height-based approach to dosing could facilitate programme implementation by removing the time and resources required to measure weight in the youngest children.^7^ Using existing data from randomised controlled trials of azithromycin distribution to children 1–59 months in Burkina Faso and Niger, we evaluated the accuracy of different age-based dosing strategies for children 1–11 months old using conservative tolerance limits. We found consistent results in both settings, suggesting a dose of 80 mg (2 mL) for children 1–2 months old and 160 mg (4 mL) for children 3–11 months old. Further optimisation for the older age group suggested high accuracy with a dose of 120 mg (3 mL) for children 3–4 months old and 160 mg (4 mL) for children 5–11 months old. Overall accuracy was similar with two-tiered and three-tiered approaches. To simplify field operations and align with the existing height poles which rely on dosing in even integers, the two-tiered approach would result in ≥89% of children receiving doses within the given tolerance limits in both settings.

The WHO guidelines on this intervention were developed in consideration of the evidence on community-based mass distribution of azithromycin available at that time, including studies of trachoma as well as the MORDOR trial.^7^ Since the release of the guidelines, several individually randomised trials focused on high-risk ill children were unable to detect effects of azithromycin on mortality.^19 20^ However, as these studies were underpowered and did not examine MDA-based interventions, comparisons with results from MDA studies are challenging. On the other hand, a long-term follow-up of azithromycin MDA in the MORDOR trial confirmed the effect of azithromycin distribution on mortality in a high mortality setting in Niger.^9^ Several high mortality West African settings are currently conducting follow-up studies and preparing programmes targeting this intervention and a simplified dosing schedule would facilitate implementation in many of these programmes. In the meantime, the WHO guidelines specify the need for additional research and will update the recommendations based on new findings. Even if this intervention is not pursued long-term for child survival, this schedule could be considered for trachoma programmes interested in examining alternatives to height-based dosing in the youngest children.

Azithromycin has a well-characterised safety profile and is typically well-tolerated in paediatric populations.^21^ Common adverse events tend to be mild and gastrointestinal in nature, including abdominal pain, vomiting, nausea, diarrhoea, dyspepsia, constipation and skin rash.^21 22^ While commonly used in children, most Food and Drug Administration indications of azithromycin are for children 6 months and older given a lack of regulatory review of safety and efficacy data for children under 6 months.^22^ The MORDOR-Niger trial included >19 500 children 1–5 months of age and found no difference in common adverse events in children receiving azithromycin compared with placebo within 2 weeks of treatment based on caregiver report.^23^ In the youngest children, observational studies have suggested that macrolides increase the risk of IHPS and may continue to increase IHPS risk up to 6 weeks of age.^17 18^ The MORDOR and CHAT studies excluded children <1 month of age to avoid potential risk in the youngest age group. However, the risk of macrolide-related IHPS in children 30–42 days old remains in question and so even slight overdosing in this age group could be a concern. No cases of IHPS were reported in the MORDOR or CHAT trials. In addition, a recent individually randomised trial of azithromycin for neonates 8–27 days old was unable to demonstrate a difference in IHPS between neonates receiving azithromycin or placebo, as only a single case of IHPS was reported in the study population of 21 832.^24^ However, determining age in days accurately can be challenging in some settings that may receive these distributions. Given these concerns, the main analyses presented here restricted the dosing for the youngest children to avoid dosing over 20 mg/kg and used more conservative tolerance limits than similar studies focused on older populations.

Age-based dosing may be less accurate than weight-based dosing given the variability of weight for a given age, yet it affords the advantage of not requiring scales and thus makes its use in mass drug administration appealing. Some mass drug administration programmes use height-based approaches to dosing as alternatives to relying on weight, including programmes distributing azithromycin for trachoma.^2 4–6^ Height typically correlates well with weight, and the trachoma programme’s existing height pole is already being used for older children in trials of azithromycin distribution for child survival. However, we found that the use of the existing height pole for children <6 months old resulted in lower accuracy compared with the age-based approach with the more conservative tolerance limits. The broader tolerance limits of 20–40 mg/kg resulted in higher accuracy using the existing height pole, which may be considered if this range is deemed appropriate for this age group. In fact, single doses up to 60 mg/kg have been shown to be well tolerated in children older than 6 months.^14^ We were able to optimise height-based dosing to improve accuracy with the conservative tolerance limits, but this would require a change to the existing height poles before implementation. Age-based dosing thus has the advantage of not requiring the use of additional tools and may facilitate faster implementation of this intervention at scale, which could result in more infant deaths averted. In addition, for young children unable to stand, height-based dosing would also necessitate additional training and two people to measure length with infants lying flat on their backs, which may be less accurate than standing height without the use of a stadiometer. On the other hand, accurate age determination can be a challenge in some settings. In the Niger trial, the majority of children’s ages were determined using caregiver report whereas in Burkina Faso, date of birth was more commonly available from health cards. Despite this difference, we saw similar distributions of ages in the two populations and the optimisation results from the two studies were well aligned, suggesting some flexibility in the accuracy required for the use of age to result in reasonable doses.

The strengths of this study include the use of large population-based datasets from two different settings. As all data were collected as part of randomised controlled trials, procedures were conducted according to standardised protocols and quality was monitored during data collection. Limitations include the lack of height data in the CHAT study, which precludes our ability to compare a height-based dosing approach across settings. In addition, the generalisability of these results is limited to similar rural settings in West Africa. Although this region is the current primary focus of studies on azithromycin for child survival, data from other high mortality settings would be valuable to validate the broader use the proposed simplified approaches.

In conclusion, this study evaluated several simplified age-based and height-based approaches to determine dose of azithromycin for children 1–11 months old and found that a two-tiered age-based approach resulted in high accuracy while balancing concern about overdosing in this young population with the simplicity of programme implementation.

10.1136/bmjgh-2022-009801.supp1 Supplementary data

## Data Availability

Data are available in a public, open access repository. The data used in the analyses presented here were generated in the MORDOR-Niger and the CHAT trials. The relevant MORDOR-Niger data are publicly available on Open Science Framework: https://osf.io/g8btp/. As the CHAT trial is currently ongoing, the baseline data are not yet publicly available and will be released with the publication of the primary outcome.
