# Supplementary material for: Simplified dosing of oral azithromycin for children 1–11 months old in child survival programmes: age-based and height-based dosing protocols
Source: BMJ Glob Health. 2022 Oct 17;7(10):e009801. doi: 10.1136/bmjgh-2022-009801 (PMC9577904; doi:10.1136/bmjgh-2022-009801)
Supplement: Supplementary data [file bmjgh-2022-009801supp002.pdf]

Supplementary Material

Supplementary Figure 1: Heat maps summarizing results of dose optimization with age cutoff determination for children 3-11 months old in the MORDOR morbidity trial.

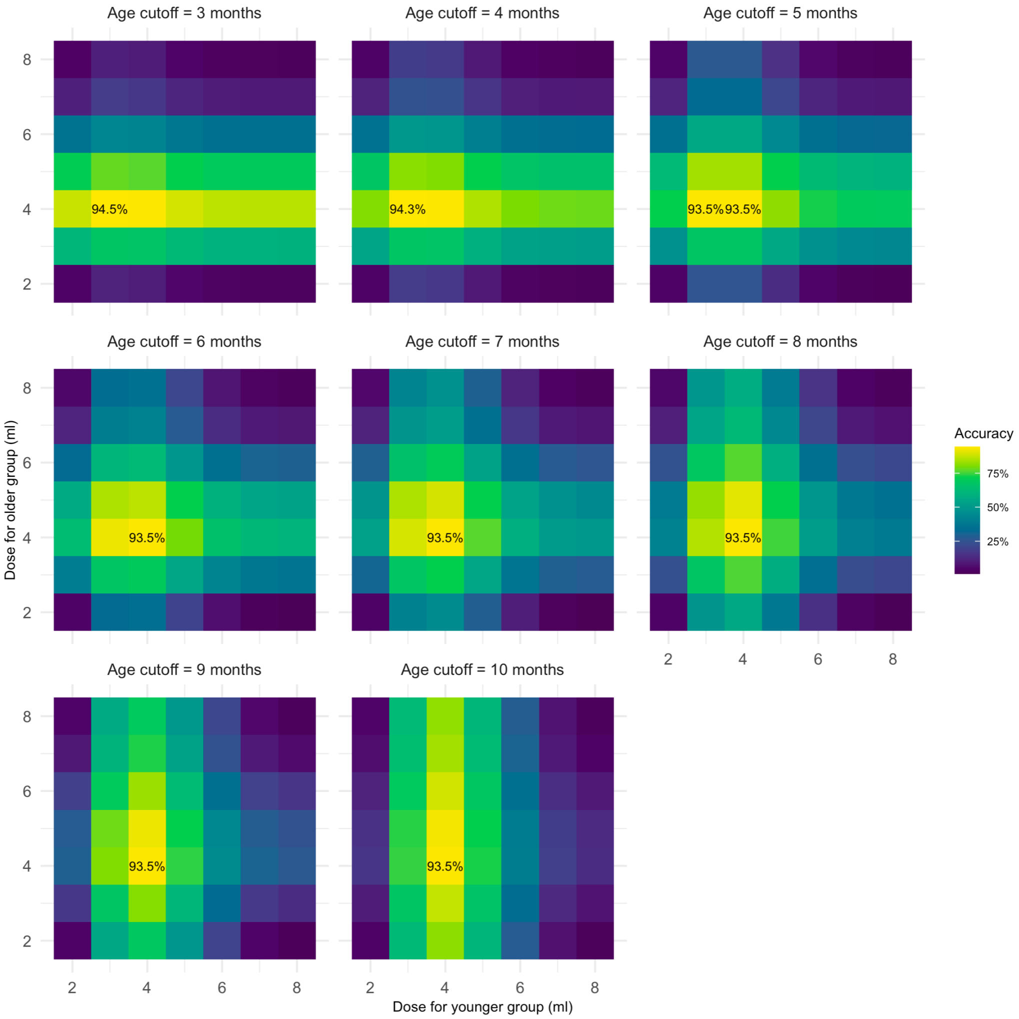

Supplementary Figure 2: Heat maps summarizing results of dose optimization with age cutoff determination for children 3-11 months old in the CHAT trial.

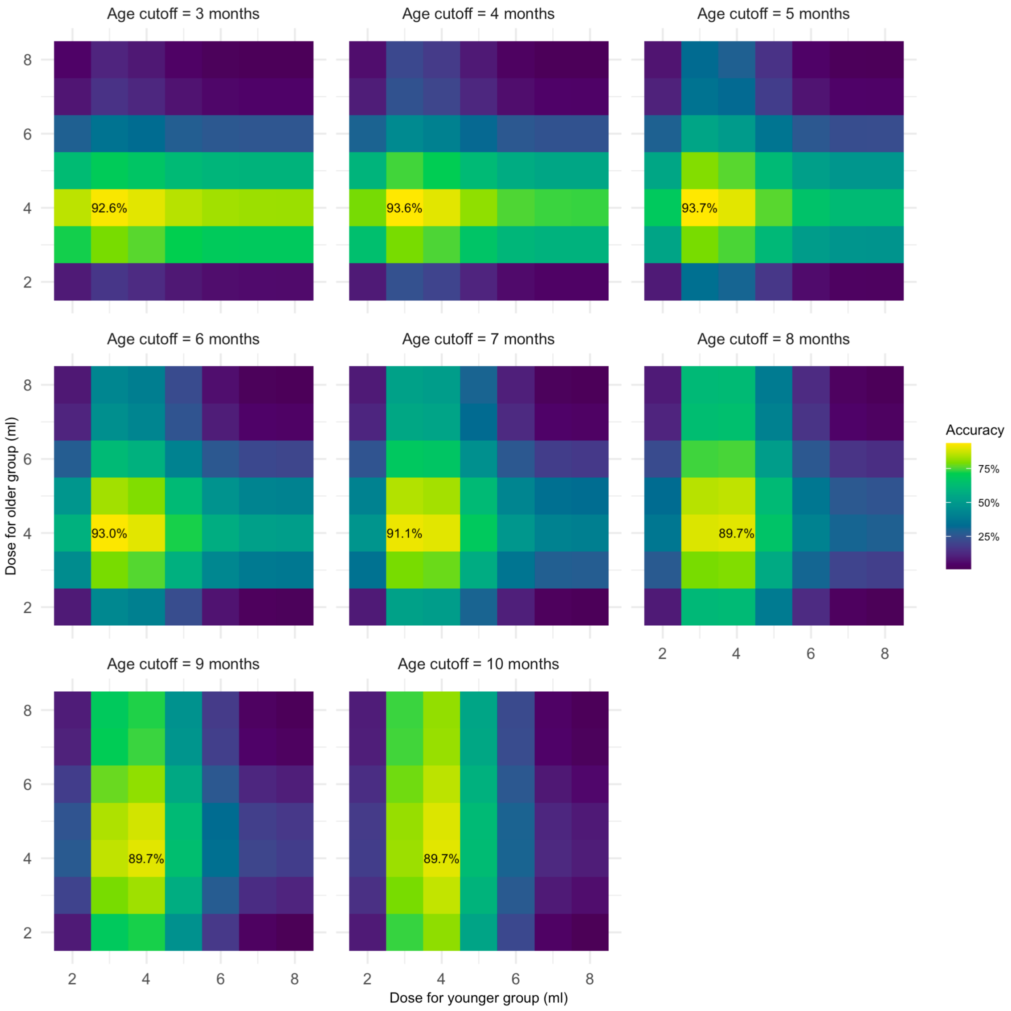

Supplementary Table 1. Sensitivity analysis for children 3-11 months old in the MORDOR and CHAT trials, without age cutoff determination and using tolerance limits of 20-40 mg/kg. Result with highest accuracy is highlighted.[1]

Study (n)	Dose	Within Limits		Underdose		Overdose		Average dose (mg/kg)
		n	%	n	%	n	%	
MORDOR (3,713)	80 mg (2 ml)	11	0.3%	3,702	99.7%	NA	NA	11.0
	120 mg (3 ml)	489	13.2%	3,224	86.8%	NA	NA	16.5
	160 mg (4 ml)	2,517	67.8%	1,186	31.9%	10	0.3%	22.1
	200 mg (5 ml)	3,478	93.7%	158	4.3%	77	2.1%	27.6
	240 mg (6 ml)	3,254	87.6%	24	0.6%	435	11.7%	33.1
	280 mg (7 ml)	2,389	64.4%	4	0.1%	1,320	35.6%	38.6
	320 mg (8 ml)	1,297	34.9%	1	0.0%	2,415	65.0%	44.1
CHAT (5,167)	80 mg (2 ml)	28	0.5%	5,139	99.5%	NA	NA	11.7
	120 mg (3 ml)	1,121	21.7%	4,046	78.3%	NA	NA	17.5
	160 mg (4 ml)	4,078	78.9%	1,080	20.9%	9	0.2%	23.3
	200 mg (5 ml)	4,855	94.0%	96	1.9%	216	4.2%	29.2
	240 mg (6 ml)	4,247	82.2%	18	0.3%	902	17.5%	35.0
	280 mg (7 ml)	2,804	54.3%	NA	NA	2,363	45.7%	40.8
	320 mg (8 ml)	1,344	26.0%	NA	NA	3,823	74.0%	46.7

mg, milligram; mg/kg, milligrams per kilogram; ml, milliliter

[1]Percentages may not sum to 100% due to rounding.

Supplementary Figure 3. Distribution of doses that would have been given to children 1-11 months in the MORDOR morbidity trial if the existing height-based dosing pole were used to determine dose. Green dashed lines indicate tolerance limits of 15-30 mg/kg and orange dashed lines indicate tolerance limits of 20-40 mg/kg.

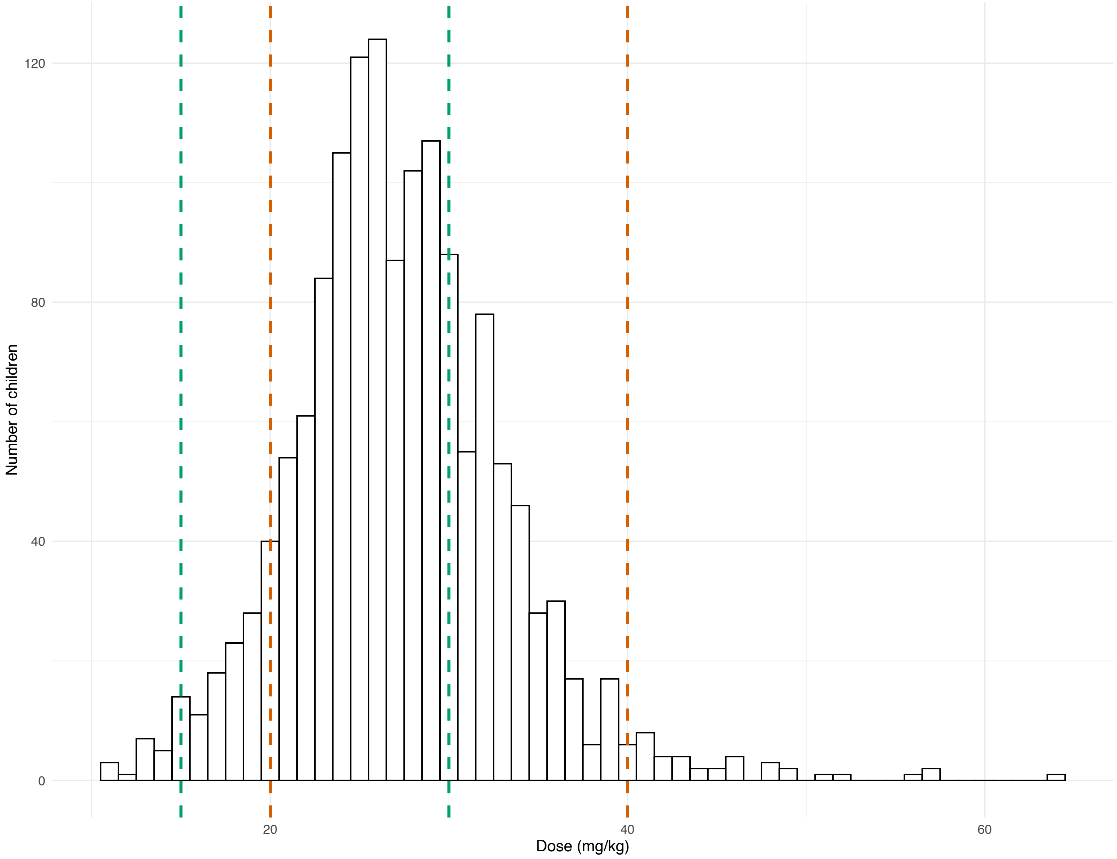

Supplementary Table 2. Top 5 results from height-based dose optimization for children 1-5 months old in the MORDOR morbidity trials (n=1405). Result with highest accuracy is highlighted for each set of analyses.[1]

Tolerance limit (mg/kg)	Height cutoff (cm)[2]	Dose 1, shorter group	Dose 2, taller group	Within Limits		Underdose		Overdose	
				n	%	n	%	n	%
15-30	60	120 mg (3 ml)	160 mg (4 ml)	1,366	94.0%	23	1.6%	65	4.5%
	65	120 mg (3 ml)	160 mg (4 ml)	1,363	93.7%	54	3.7%	37	2.5%
	65	120 mg (3 ml)	200 mg (5 ml)	1,322	90.9%	45	3.1%	87	6.0%
	70	120 mg (3 ml)	160 mg (4 ml)	1,322	90.9%	102	7.0%	30	2.1%
	70	120 mg (3 ml)	200 mg (5 ml)	1,321	90.9%	95	6.5%	38	2.6%
20-40	60	160 mg (4 ml)	200 mg (5 ml)	1,388	95.5%	27	1.9%	39	2.7%
	65	160 mg (4 ml)	200 mg (5 ml)	1,365	93.9%	58	4.0%	31	2.1%
	65	160 mg (4 ml)	240 mg (6 ml)	1,357	93.3%	46	3.2%	51	3.5%
	55	120 mg (3 ml)	200 mg (5 ml)	1,333	91.7%	35	2.4%	86	5.9%
	55	160 mg (4 ml)	200 mg (5 ml)	1,327	91.3%	18	1.2%	109	7.5%

cm, centimeter; mg, milligram; mg/kg, milligrams per kilogram; ml, milliliter

[1]Percentages may not sum to 100% due to rounding.
[2]Height cutoffs refer to the height at which the shorter group ends. For example, a height cutoff of 60 indicates a shorter group of ≤ 60 cm and a taller group of > 60 cm.
