# Supplementary material for: Simplified dosing of oral azithromycin for children 1–11 months old in child survival programmes: age-based and height-based dosing protocols
Source: BMJ Glob Health. 2022 Oct 17;7(10):e009801. doi: 10.1136/bmjgh-2022-009801 (PMC9577904; doi:10.1136/bmjgh-2022-009801)
Supplement: Supplementary data [file bmjgh-2022-009801supp001.pdf]

Reflexivity Statement

1. **How does this study address local research and policy priorities?**

The aim of this study is to identify simplified approaches do oral azithromycin dosing to facilitating programmatic implementation of a child survival intervention. This intervention is being explored by our research partners and other relevant policy stakeholders in Burkina Faso and Niger and the question of how to transition from trial to program is of significance to local teams. One of the big questions raised by our partners is how to simplify dosing to increase the feasibility of field operations.

2. **How were local researchers involved in study design?**

The first category of local researchers involved were those with extensive involvement in conducting, leading, and organizing the main trials from which the data used in this study were generated (AMA, AA, RM, AKM in Niger and AS, MO, MB, VB in Burkina Faso). The second category of local researchers includes those who are engaged in another project in Burkina Faso for which this research question is of vital importance (FY-W, IB in Burkina Faso). The third category of researchers are those working for institutions based in high income country settings that support programs and research in this content area (CAE from Pfizer, PE and PJH from International Trachoma Initiative). The final group of researchers include the US-based team involved in the design and conduct of the main trials as well as the analysis for the data for the present project (HH, EL, JB, FN, CEO, TML, and KSO).

3. **How has funding been used to support the local research team?**

The present project is unfunded, but the main trials that generated the data used in the present project were funded by the Bill & Melinda Gates Foundation and the funds were split between the local research team and the US-based team.

4. **How are research staff who conducted data collection acknowledged?**

Key contributors to data collection are included as authors.

5. **Do all members of the research partnership have access to study data?**

All members of the partnership have access to data.

6. **How was data used to develop analytical skills within the partnership?**

The present project was not directly used to develop analytical skills within the partnership, but the main trials on which this project is based both include analytic projects led by local partners.

7. **How have research partners collaborated in interpreting study data?**

All partners involved in this project contributed to the review and interpretation of study data.

8. **How were research partners supported to develop writing skills?**

The writing for this project was led by the US-based team and although writing skills were not a focus of the present project, the main trials on which this project is based both include writing projects led by local partners with support from the US-based team.

9. How will research products be shared to address local needs?

The results of this project will be shared directly with partners and stakeholders and be included for consideration of the development of this intervention in West African countries considering its implementation.

10. How is the leadership, contribution and ownership of this work by LMIC researchers recognised within the authorship?

The authorship list is split evenly between the local partner collaborators and the US-based researchers. The first and last author positions are held by members of the US-based research team, as they led the development of the question, the analysis, and the writing for this project.

11. **How have early career researchers across the partnership been included within the authorship team?**

KSO, AMA, AKM, MO, MB, and VB are each early career researchers involved in this project who have been included in the authorship team.

12. How has gender balance been addressed within the authorship?

11 authors are men (AMA, AS, AA, AKM, MO, MB, VB, IB, CAK, PE, TML) and 9 authors are women (HH, RM, FY-W, EL, JB, FN, CEO, KSO)

13. How has the project contributed to training of LMIC researchers?

Several LMIC researchers on the authorship team are in the process of completing doctoral level training (AMA, MO, MB) involving projects related to the main trials.

14. How has the project contributed to improvements in local infrastructure?

This project has not directly contributed to improvements in local infrastructure.

15. What safeguarding procedures were used to protect local study participants and researchers?

There was no primary data collection as part of this project, therefore this question is not directly applicable.
